# Taxonomic revision of *Disporum* Salisb. (Colchicaceae, Uvularioideae) of Taiwan

**DOI:** 10.3897/phytokeys.119.33516

**Published:** 2019-03-15

**Authors:** Chao Chien-Ti, Tseng Yen-Hsueh

**Affiliations:** 1 School of Life Science, National Taiwan Normal University, No. 88, Tingchou Rd. 4 section, Wenshan district, Taipei city 116, Taiwan National Taiwan Normal University Taipei Taiwan; 2 Department of Forestry, National Chung Hsing University, No. 145, Xinda Rd., South district, Taichung city 402, Taiwan National Chung Hsing University Taichung Taiwan

**Keywords:** *
Disporum
*, Colchicaceae, plant taxonomy, Taiwan

## Abstract

A taxonomic revision of *Disporum* of Taiwan is presented with two species and one variety being recognised. The diagnostic characters of *Disporum* include the colour of tepals, stolon morphology, the trichomes of filaments and style and leaf morphology. These characters, along with karyotype and pollen morphology, are discussed and evaluated amongst different taxa. As a result, *D.kawakamii* and *D.shimadae* are treated as independent species, rather than varieties of *D.cantoniense* and *D.sessile*, respectively. *Disporumnantouense* is treated as a synonym of D.sessilevar.intermedium**stat. nov.** Detailed descriptions, type information, diagnostic key, line drawings, photos and distribution maps are provided.

## Introduction

*Disporum* Salisb. comprising of about twenty species, is distributed from the Himalayas through Vietnam, China, Japan, Korea and Russia. More than fourteen species have been found in China (Chen et al. 2000), four species are recorded in Japan (Tamura 2016) and four species are recorded in Taiwan (Ying 2000). Some new species have recently been described (Hu et al. 2016; Hareesh et al. 2018). This genus is characterised by having fleshy roots, erect stems with scale leaves at the lower part, pendulant flowers and berries, diagnostic characters of intrageneric taxa dependent on whether stolons are present, tepals spreading or not, colour and shape of tepals, length and trichome type of filaments and styles and colour of fruits (Hara 1988; Chen et al. 2000).

Taiwanese species have been studied by several authors from a morphological and karyological standpoint (Matsumura and Hayata 1906; Kawakami 1910; Hayata 1911; Chao et al. 1963; Liu and Ying 1978; Hara 1988; Ying 1989; Ying 1990ab; Wang 1997; Ying 2000), but each author had his/her own way of treating different ranks. Chao et al. (1963) recognised two species, *D.kawakamii* and *D.shimadae*, Liu and Ying (1978) three species, *D.kawakamii*, *D.pullum* Salisb. & Hook. f., and *D.shimadae* and Hara (1988) recorded two varieties and one form, D.cantoniense(Lour.)Merr.var.kawakamii (Hayata) Hara, D.sessile(Thunb.)D. Don & Schult.var.shimadae (Hayata) Hara and D.sessilevar.shimadaef.intermedium Hara. Ying (2000) treated the genus as consisting of four species *D.kawakamii*, *D.nantouense* Ying, *D.shimadae* and *D.taiwanense* S. S. Ying.

Recently, in studying Liliaceae s. l. of Taiwan, we found that the diagnostic characters of *Disporum* were highly variable; moreover, the taxonomy of the species occurring in Taiwan has been confused by different authors. Therefore, we deemed that a revision of *Disporum* of Taiwan was necessary. In this study, we revise the genus by reviewing the literature and examining type specimens in herbaria, along with comparisons of the morphology, chromosome number and pollen morphology of specimens and establishing their distribution.

## Materials and methods

The study materials were collected from the field and from herbarium specimens. Living plants were cultivated in the greenhouse of the Department of Forestry, NCHU. All voucher specimens were deposited in the herbarium of the Department Forestry, National Chung Hsing University (TCF). Specimens from the following herbaria specimens were examined: CHIA, HAST, KYO, PPI, TAI, TAIE, TAIF and TI (acronyms following Thiers, 2019, continuously updated).

### Pollen morphology

Pollen was collected from fresh anthers of flowers in anthesis. Voucher specimens (Table 1) were deposited in TCF. The pollen samples were fixed in 70% ethanol (EtOH) and serially dehydrated with 80%, 90%, 95%, 99.5% EtOH and lastly with acetone. After drying with the Quorum E3100 critical-point dehydrator, the pollen specimens were observed and photographed with a scanning electron microscope (HITACHI S-3400N). Descriptions of pollen morphology used the terminology of Punt et al. (2007) and Hesse et al. (2009).

**Table 1.** Pollen materials of *Disporum* in this study.

Taxa	Coll. location	Coll. no.
* D. kawakamii *	Hualien: Yenhai forest track	Chao 1376
D. sessile var. intermedium	Hsinchu: Yuanyang lake	Chao 1400
* D. shimadae *	Keelung: Tawulun fort	Chao 1285

### Distribution and conservation rank rvaluation

The distribution of each taxon was determined from the data on herbarium sheets and from our own field records. Only recognised specimens were marked on the map. The conservation rank evaluation followed the protocols of The Red List of Vascular Plants of Taiwan, 2017 (editorial committee of The Red List of Vascular Plants of Taiwan 2017).

## Results

### Taxonomic diagnostic characters

**Habit**

The habit of *Disporum* in Taiwan includes two types, evergreen and deciduous. The evergreen type includes only *D.kawakamii* and the deciduous type includes two other taxa. The above-ground part of the deciduous types dies back in winter, leaving a dormant bud above the roots. The bud sprouts in the spring and grows up to form a new stem. The evergreen species produces similar buds at the same position, but the above-ground parts do not die back in winter and last into the next year.

**Roots and stolons**

The roots of *Disporum* are fleshy and glabrous without root hairs, which is a diagnostic character of the genus. Amongst the Taiwanese species, stolons are present in D.sessilevar.intermedium and *D.shimadae*, but absent in *D.kawakamii*. The stolon is subterranean and creeping and the terminal bud becomes the new individual plant.

**Leaves**

The leaves of *Disporum* are simple, alternate, subsessile, glabrous and with an entire margin and stipules. The shape could be classified into two types, lanceolate to linear-lanceolate and elliptic to oblong. The former includes D.sessilevar.intermedium and *D.shimadae* and the latter *D.kawakamii*. Although the leaf shape of *D.kawakamii* is often elliptic to oblong, narrower leaves could be found on some individuals.

**Inflorescence and flowers**

The inflorescences of *Disporum* are pseudo-terminal and umbellate, with pendulous flowers. The flower is comprised of six tepals, six stamens and a pistil. The tepals are variable in shape and have a short gibbous spur and nectary. They are variable in colour, but in Taiwan are white, yellow or variable in colour. White tepals are found in D.sessilevar.intermedium, yellow in *D.kawakamii* and *D.shimadae*. The apices of the tepals of D.sessilevar.intermedium often have purplish or reddish spots, respectively. Some populations of *D.kawakamii* have deeper colouration and these had been described as a new species, *D.taiwanense*, based on this character; but the colour can vary with the habitat: the more exposed location it is growing in, the more sunlight and the deeper the colour and vice versa.

**Pollen morphology**

**Figure 1.**
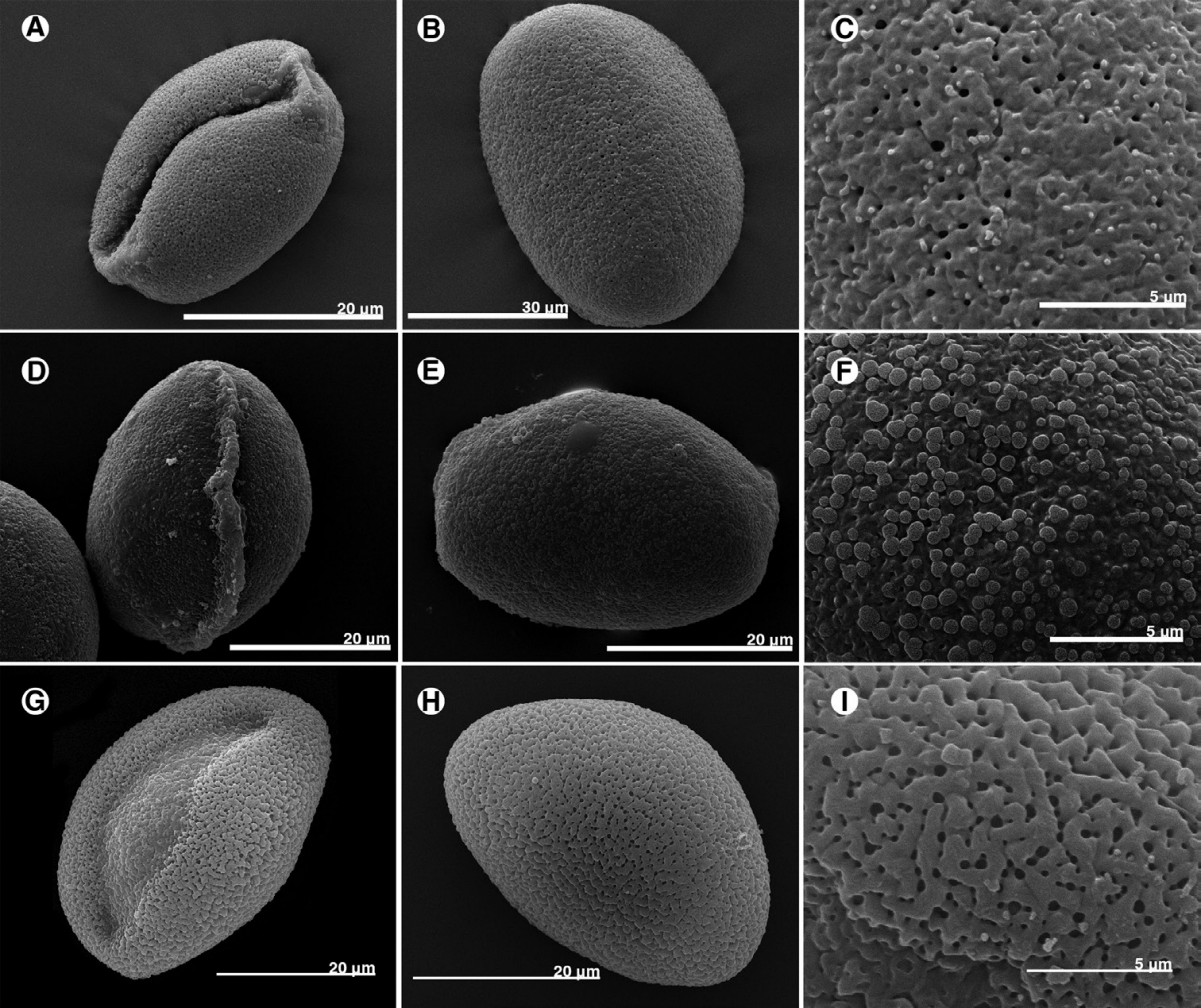
Pollen morphology of *Disporum* of Taiwan. **A−C***D.kawakamii***D−F**D.sessilevar.intermedium**G−I***D.shimadae*. **A, B, D, E, G, H** polar view **C, F, I** sculpture.

The pollen grains of *Disporum* taxa of Taiwan are medium-sized monads, monosulcate, prolate, with the length of the equatorial and polar axes being 36.57–44.76 µm and 26.77–28.91 µm, respectively. Sculptural type varies amongst taxa: *D.kawakamii* and D.sessilevar.intermedium are foveolate and areolate, while *D.shimadae* is rugulate. Huang (1972) studied the pollen morphology of *D.kawakamii* and *D.shimadae* by light microscopy and his results showed that the pollen grains of two species were monads, monosulcate, subspheroidal and the sculpture was reticulate. Most of his results are similar to ours, but he sometimes recognised different sculptural types. The different types of microscopy used may lead to the different results (Fig. 1) (Table 2).

**Table 2.** Pollen morphology of Disporum of Taiwan.

	*** D. kawakamii ***	** D. sessile var. intermedium **	*** D. shimadae ***
polar axis (µm)	29.12±0.97	26.90±0.20	27.41±1.32
equatorial axis (µm)	43.31±2.02	37.45±3.30	39.77±2.21
P/E	0.67±0.02	0.78±0.08	0.69±0.03
shape	oblate	oblate	oblate
size	medium	medium	medium
aperture	1-sulcate	1-sulcate	1-sulcate
sculpture	foveolate	areolate	rugulate

**Chromosome number**

The chromosome number of *Disporum* of Taiwan had been determined by Chuang et al. (1962), Chao et al. (1963), Hsu (1971), Chang and Hsu (1974), Tamura et al. (1992) and Wang (1997). The results revealed that *D.kawakamii* and D.sessilevar.intermedium had 2n = 16 chromosomes, while Hsu (1971) reported tetraploidy (2n = 32) for *D.kawakamii*. *Disporumshimadae* was reported as 2n = 14 in the studies of Tamura et al. (1992) and Wang (1997), but Chang and Hsu (1974) reported the chromosome number as 2n = 16 (Table 3).

**Table 3.** Previous chromosome count of Disporum of Taiwan.

Taxon	Chromosome number		Reference
	n	2n	
* D. kawakamii *	8		Chuang et al. 1962
		16	Chao et al. 1963; Tamura et al. 1992; Wang 1997
		32	Hsu 1971
D. sessile var. intermedium		16	Tamura et al. 1992 (as *D.taipingense*); Wang 1997 (as *D.nantouense*)
* D. shimadae *		14	Chao et al. 1963; Chang and Hsu 1974; Tamura et al. 1992; Wang 1997
		16	Chang and Hsu 1974

**Distribution**

**Figure 2.**
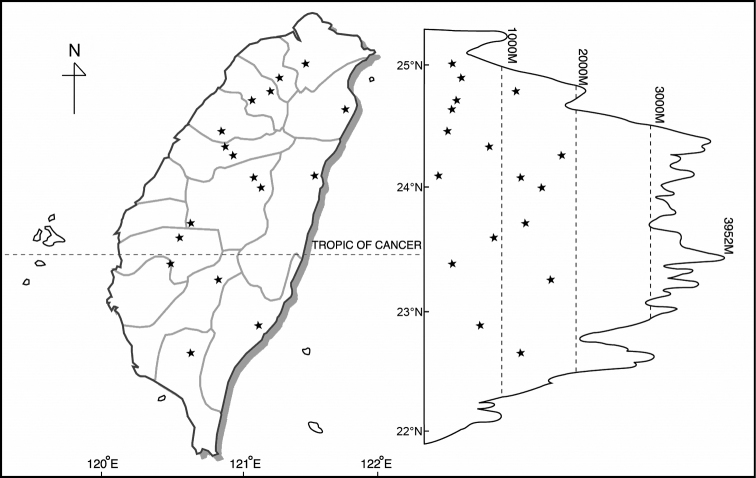
Distribution of *Disporumkawakamii*.

**Figure 3.**
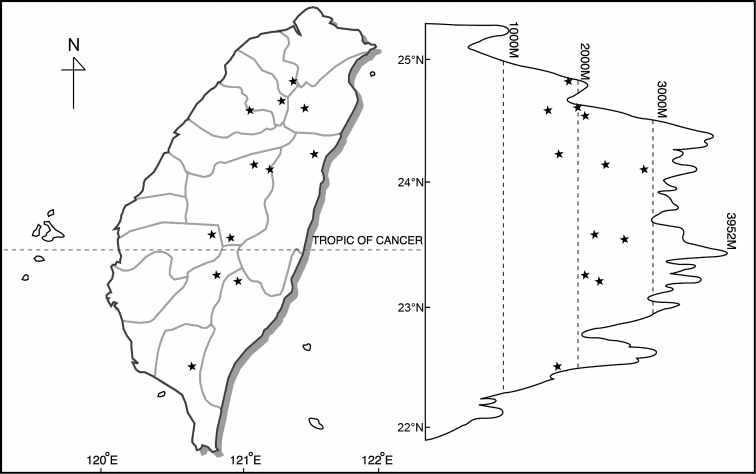
Distribution of Disporumsessilevar.intermedium.

**Figure 4.**
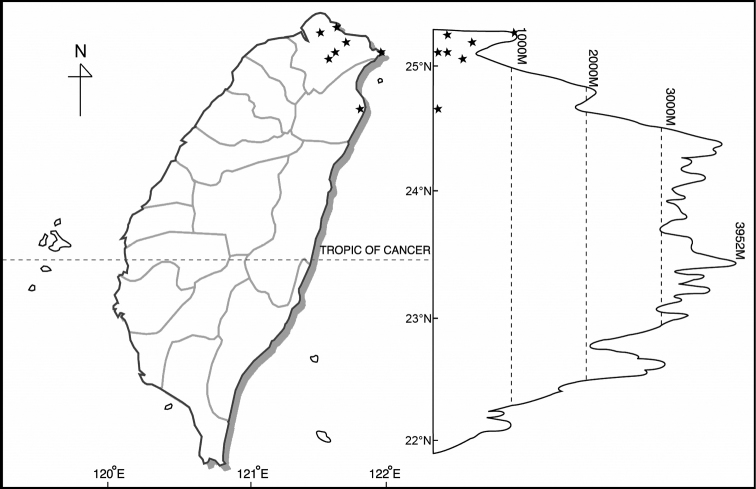
Distribution of *Disporumshimadae*.

*Disporum* taxa are found from near sea level to about 2900 m in the mountainous regions of Taiwan, but each species has a different distribution pattern. *Disporumkawakamii* is found in low to medium altitude mountain areas, often grows as an understorey plant in a forest or at the edge of a forest, but sometimes even appears on an exposed roadside. Disporumsessilevar.intermedium is found primarily at medium altitudes, from 1500 m to 2900 m in moist and shady areas of forest. *Disporumshimadae* is only found in the north-eastern part of Taiwan, from sea level to low-lying mountains or hills (Table 4) (Figs 2–4).

**Table 4.** Comparisons of Disporum species of Taiwan.

	* D. kawakamii *	D. sessile var. intermedium	* D. shimadae *
habit	evergreen	deciduous	deciduous
stolon	absent	present	present
leaf shape	elliptic to oblong	linear-lanceolate	linear-lanceolate
color of tepal	pale-yellow with reddish apex	white with green base and apex	yellow
distribution	low to medium altitude mountain area	medium altitudes ca. 1500−2900m	north-eastern part, seashore to low altitude mountains

**Evaluation of conservation rank of *Disporum***

According to a study by the editorial committee of The Red List of Vascular Plants of Taiwan (2017), the conservation rank of *D.kawakamii* and *D.shimadae* was least concern (LC), whereas *D.nantouense* (= D.sessilevar.intermedium) and *D.taiwanense* (= *D.kawakamii*) were data deficient (DD). The results of our study were similar, but we determined that the rank of D.sessilevar.intermedium was LC. This was a poorly know taxon, the specimens often being misidentified as another species. During our field survey, we found many populations at a medium altitude, in good habitats with little disturbance. Therefore, we suggest changing the conservation rank of D.sessilevar.intermedium from DD to LC.

## Discussion

### Comparison of *D.cantoniense* and *D.kawakamii*

*Disporumcantoniense* is a widely distributed species, found from the Himalayas of northern India to Sikkim, Burma, Thailand, Vietnam and China. Hara (1988) listed four varieties of this species: one of them was D.cantoniense(Lour.)Merr.var.kawakamii (Hayata) Hara. The main characters of this variety include glabrous pedicels, apices of tepals obtuse or short acute and densely papillate (Hara 1988). Our observations show that, except for D.cantoniensevar.kawakamii, other varieties had acuminate to acute tepals somewhat reflexed when in anthesis. D.cantoniensevar.kawakamii was also the only variety with yellow tepals; the others had green, purple or purplish-brown tepals. The karyological study of Tamura et al. (1992) suggested different karyotypes for *D.cantoniense* (type A and C3) and *D.kawakamii* (type D). Molecular studies (Shinwari et al. 1994; Tamura et al. 2013) also revealed that the two species were placed in different clades. In conclusion, *D.kawakamii* is different from *D.cantoniense* on morphological, karyological and genetic levels and should be treated as an independent species rather than a variety as Hara (1988) published.

Ying (1989) described a new species, *D.taiwanense*, from eastern Taiwan that was distinguished by its usually reddish petioles and yellow tepals with reddish tips, especially on the inner surface. As we mentioned before, the colour of tepals can vary depending on the habitat conditions; therefore, this character would seem to be a poor one for distinguishing them. Since the distribution of the two taxa is also similar, we chose to consider *D.taiwanense* as a synonym of *D.kawakamii*.

### The taxonomic status of *D.nantouense* and *D.shimadae*

*Disporumnantouense* has been treated as a form D.sessilevar.shimadaef.intermedium (Hara 1988) or as an independent species (Ying 1990a). These treatments were evaluated and considered, but none of them was found suitable for this taxon. The diagnostic characters of this taxon include lanceolate to narrow lanceolate leaves, white tepals, sub-papillose filaments and chromosome number of 2n = 16. These characters are different from those of *D.shimadae*, therefore, this taxon should not be considered simply as another form of *D.sessile* as Hara (1988) proposed. These specific characters are, however, found in *D.sessile* and the molecular study of Tamura et al. (2013) also suggested close affinity between them. Thus, the proposal of Ying (1990a) that this is an independent species lacks sufficient evidence.

Tamura (2016) distinguished two varieties of *D.sessile*, namely *D.sessile* D. Don *ex* Schult. var. micranthum Hatsu. *ex* M. N. Tamura & M. Hotta and *D.sessile* D. Don *ex* Schult. var. minus Miq. The Taiwanese taxon resembled D.sessilevar.micranthum in that they both had small flowers (1.5 to 2.2 cm in length) and sub-papillose filaments, but otherwise they were distinguished by the subterranean stolons, lanceolate to narrow lanceolate leaves and tepals greenish at the apex. Based on the study of Tamura et al. (2013) and Tamura (2016), the Taiwanese taxon was merged into the monophyletic group of *D.sessile*, with the different morphological characters mentioned above. Thus, we treated this taxon as a variety of *D.sessile*, rather than a species or a form of *D.sessile*.

*Disporumshimadae* was first described by Hayata (1911) as a separate species, but Hara (1988) considered it a variety of *D.sessile*. Although the two taxa have similar morphological characters, such as leaf shape, long creeping stolons and deciduous habit, *D.shimadae* could be distinguished by its yellow flowers, thicker leaves and glabrous filaments. Furthermore, as mentioned above, the chromosome number of *D.shimadae* is 2n = 14, while that of *D.sessile* is 2n = 16 (Tamura et al. 1992; Wang 1997). This evidence would imply they are not conspecific, similar to the results of the molecular study of (Tamura et al. 2013); consequently, we chose to treat *D.shimadae* as an independent species, not a variety of *D.sessile*.

### The status of *D.taipingense*

Another taxon, related to D.sessilevar.intermedium, was *D.taipingense* M. N. Tamura & S. Kawano, nom. nud. This name had been cited in the studies of Tamura et al. (1992) and Shinwari et al. (1994), where they stated that this species was collected from Mt. Taipingshan, at an altitude of 1900 m in a coniferous forest but provided limited morphological description. They cited an article, in press, that could possibly be the original publication of the name (Tamura and Kawano 1994, Biosystematic Studies in *Disporum*, Liliaceae-Polygonateae V., A Taxonomic Revision of Species in Taiwan in Acta Phytotaxonomica et Geobotanica), but we could not find the article in the journal or the description and the type specimen. Thus, this name is *nomen nudum* due to its invalid publication. *Disporumtaipingense* was treated as a synonym of *D.nantouense* in Flora of China (Chen et al. 2000). In consideration of the facts that the collectors of the plant had deposited the specimens labelled as *D.taipingense* by M. N. Tamura in the TI herbarium and the plant materials were from Mt. Taipingshan, but morphological descriptions were inadequate and the “in press” journal article could not be found, we chose to treat this taxon as a synonym of D.sessilevar.intermedium.

According to the results, the following taxonomic treatments are made:

### Taxonomic treatment

#### Key to *Disporum* species in Taiwan

**Table:** 

1	Plant deciduous, with stolon; leaves lanceolate to linear-lanceolate	**2**
–	Plant evergreen, without stolon; leaves elliptic to oblong	**1. *D.kawakamii***
2	Tepals yellow, without spots	**3. *D.shimadae***
–	Tepals white, often with purplish spots, apex green	**2. D.sessilevar.intermedium**


### *Disporum* Salisb. *ex* D. Don, Prodr. Fl. Nepal. 50. 1825

Perennial herbs, often with short rhizome, sometimes with long creeping stolon, often glabrous, sometimes scabrous. Roots fleshy, glabrous. Stem erect, simple or branched at the upper part, with scale leaves at the lower nodes, persistent. Leaves evergreen or deciduous, simple, alternate, sessile or subsessile, linear to suborbicular, 3−several-nerved, estipule. Inflorescences pseudoterminal, solitary to umbel, bract absent. Flowers bisexual, actinomorphic, pendulate to spread, tepals 6, free, 2-whorled, subequal, white, green, yellow or purple, often saccate or spurred at basal part. Stamen 6, inserted at base of tepals. Filaments usually slightly flattened, glabrous of papillose. Anthers basifixed, to innate, extrorse, 2-loculed. Ovary superior, 3-loculed, ovules 2−6 per locule. Style straight, 3-lobed to 3-fid apically. Fruits berry, dark purple to black, 2−6 seeds.

About 20 species, from Himalaya region, India, Myanmar, Bhutan, Sikkim, Thailand, Vietnam, China, Taiwan, Japan to Korea.

#### 1. Disporum kawakamii

Taxon classificationPlantaeLilialesColchicaceae

Hayata, J. Coll. Sci. Imp. Univ. Tokyo 30(1): 365. 1911

Figs 5 , 6 臺灣寶鐸花

Disporum kawakamii Hayata, J. Coll. Sci. Imp. Univ. Tokyo 30(1): 365. 1911. Hayata, Gen. Ind. 85. 1917; Sasaki, List Pl. Form., 106. 1928; Masamune and Simada, Short Fl. Form. 269. 1936; Masamune, List Vasc. Pl. Taiwan. 132. 1954; Chao et al., Bot. Bull. Acad. Sin. New Series 4(2):81; Ying, The Liliaceae of Taiwan. 25. 1969; Wang, Cytotaxonomy of Liliaceae in Taiwan (II) Polygonateae and Tricyrteae. 41. 1997; Liu & Ying, Fl. Taiwan. 5:52. 1978; Ying, Liliaceae of Taiwan. 27. 1990; Chen et al., Fl. China 22:157, 2000; Ying, Fl. Taiwan 2nd ed. 5:44. 2000; Boufford et al., Fl. Taiwan 2nd ed. 6:111. Lectotype: Kagi, Suitoryo, 20 Mar. 1908, T. Kawakami 3493. (lectotypification: Hara, 1988) (TI!) Syntype: in monte Morrison, 13 Oct. 1906, T. Kawakami & U. Mori 1726. (TI)
Disporum cantoniense (Lour.) Merr. var. kawakamii (Hayata) Hara, Univ. Mus. Univ. Tokyo Bull. 31:187. 1988.
Disporum pullum auct. non. Salisb. *ex* Hook. f.: Matumura & Hayata, J. Coll. Sci. 1Imp. Univ. Tokyo 22: 443. 1906.
Disporum taiwanense S. S. Ying, J. Jap. Bot. 64(5):151. 1989. Ying, Liliaceae of Taiwan. 29. 1990; Ying, Fl. Taiwan 2^nd^ ed. 5:46. 2000; Boufford et al., Fl. Taiwan 2^nd^ ed. 6:111. Type: Hualien county, Taroku to Talishih, 4 Apr. 1988, Ying s. n. (Holotype: NTUF!)

##### Perennial herbs.

Stem erect, up to 1.3 m, branched at the upper part, lower nodes covered with persistent scale leaves. Leaves evergreen, simple, alternate, elliptic to oblong, 6−8 cm long, 3−6 cm wide, 3−5 nerved, apex acuminate, base attenuate, petiole short, 3−5 mm long, green, sometimes reddish, glabrous, estipulate. Inflorescence pseudoterminal, solitary to 3−5 flowers fascicled, peduncle short, 3−5 mm long, bract absent. Tepals 6, arranged into 2-whorls, spathulate, 1.5−2 cm long, 5−8 mm wide, light yellow to yellow, often with reddish spots near apex, papillate at the base of the inner surface and margin, 3-nerved, base with a short spur, 1−2 mm long, nectary inside. Stamens 6, inserted at the base of tepals, filaments ca. 1 cm long, expansion at the lower part, slightly papillose at proximal part, anthers 2-loculed, basifixed, ca. 3 mm long, longitudinally dehiscent. Ovary superior, ovate to orbicular, ca. 3 mm long, 3-loculed, style slender, 7−9 mm long, stigma 3-fid, pubescent. Fruits berry, globose or compress globose, 6−7 mm long, 4−6 mm in diam., purplish-black. Seeds numerous. 2n = 16.

**Figure 5.**
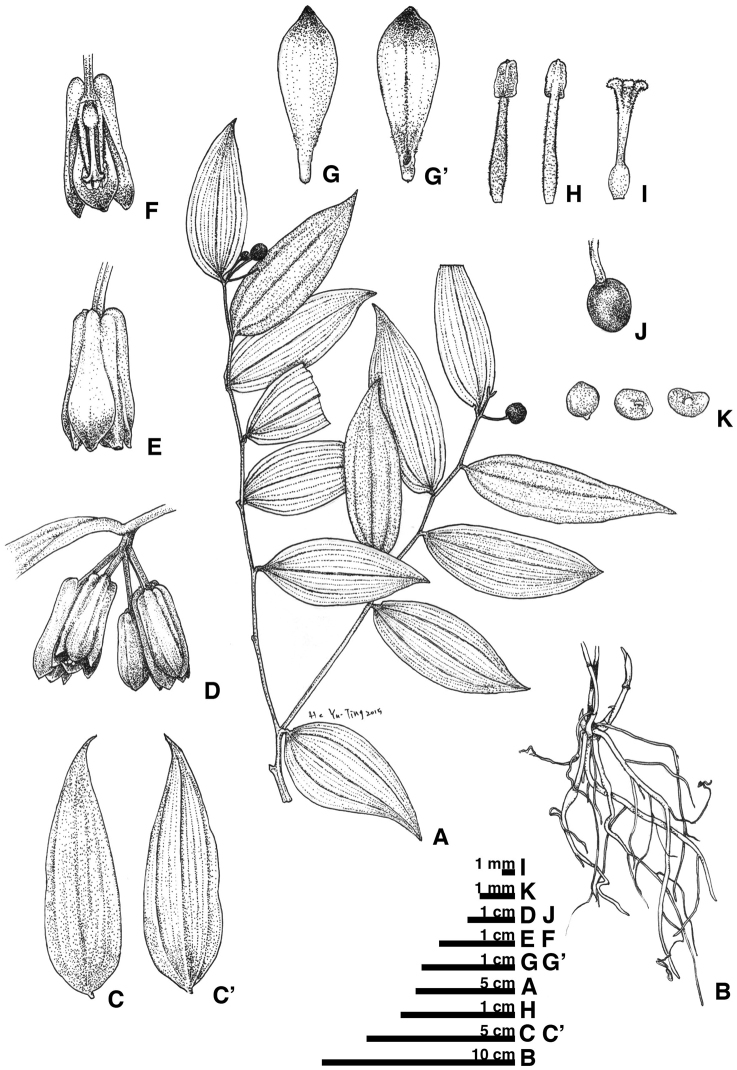
*Disporumkawakamii*. **A** habit **B** underground part **C** leaf adaxial surface **C**’ leaf abaxial surface **D** inflorescence **E** flower **F** flower section (with tepals and stamen removed) **G** tepal outer surface **G**’ tepal inner surface **H** stamen **I** pistil **J** fruit **K** seeds.

**Figure 6.**
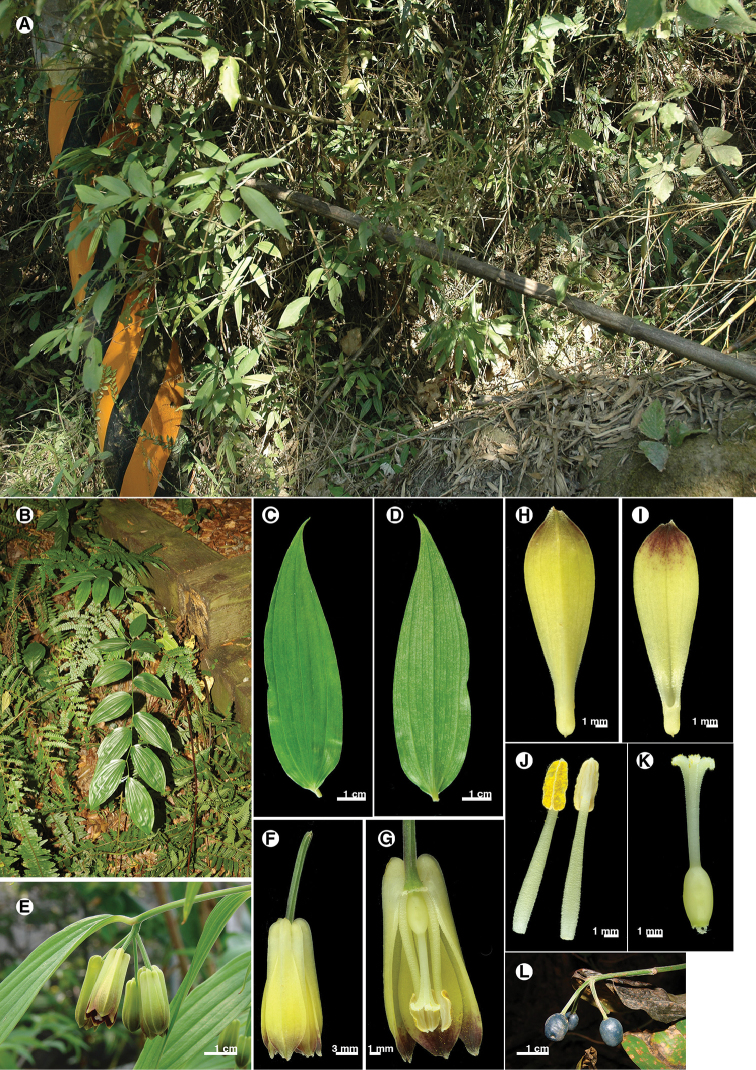
*Disporumkawakamii*. **A** habitat **B** habit **C** leaf adaxial surface **D** leaf abaxial surface **E** inflorescence **F** flower **G** flower section (with tepals and stamen removed) **H** tepal outer surface **I** tepal inner surface **J** stamen **K** pistil **L** fruits.

##### Endemic to Taiwan.

Distributed in low to medium altitude mountains up to 2,000 m.

##### Additional specimens examined.

Chiayi county: Alishan-Fengshan, 8 Sep 1993, C. M. Wang W00265 (TNM); Yuntan Rest Station-Shuisheliao, 10 Sep 2004, C. M. Wang & Y. M. Hsu 7730 (TNM); Tatienyupupu, 28 Oct 1997, M. Y. Shen 1980 (TAIE); en route from Chiehtung “Villa” to Tienyunshan, 850 m alt., 1 Nov 1985, C. I Peng 8873 (HAST); Juili, 860 m alt., 26 Jan 1997, T. Y. A. Yang et al. 7838 (PPI); Hsinchu county: Kenliaoping, 26 Apr 1994, C. M. Wang W00733 (TNM); on the way from Shihlu to Chingchuan, 27 Jul 1994, K. Y. Wang & T. Y. Liu 75 (TNM); Niaotsui, 21 Oct 2001, C. M. Wang & C. Y. Li 5300 (TNM); Shakaro ancient trail, 1500−1700 m alt., 10 May 2016, P. H. Chen 1096 (PPI); Luoshan forest track, 1000 m alt. 20 Dec 2002, Y. Y. Huang 1309 (HAST); Hualien county: Sanchan, 7 Sep 1996, C. K. Liou et al. 539 (TNM); Yenhai-lindao, 3 Apr 1991, T. Y. Yang et al. 5506 (TNM); Mt. Paliwanshan, 28 Jul 2007, C. H. Chen et al. 8339 (TNM); Shakadang forest road 2 km, 30 Mar 2008, T. Y. A. Yang et al. 20106 (TNM); Huitouwan, 9 Dec 1983, T. C. Huang & S. F. Huang 10200 (TAI); Lanshan, 1900 m alt. 16 Aug 1967, S. C. Hsu 3539 (TAI); Yushan National Park Nanan Recreation Center to Walami, 365 m alt., 16 Apr 1995, H. Y. Shen 683 (HAST); Mukuashan, 700−1000 m alt., 16 Apr 2003, P. J. Lin et al. 159 (HAST), Taorko to Tali, 15 Jun 2007, P. F. Lu 13967 (HAST); Ilan county: Mingchih-Chilan, 15 Apr 2002, C. M. Wang 5468 (TNM); Taipingshan, 5 Apr 2005, C. H. Wu et al. 343 (TNM); Chunchien, 1500–1600 m alt., 30 Jul 1996, Y. C. Chen et al. 17 (HAST); Ssuchi forest road, 1360 m alt., 18 Jul 1999, C. I Huang 399 (HAST). Nanao, 28 Apr 2001, W. F. Ho 1215 (TNM); Mt. Tuli, 450 m alt., 31 Mar 1985, S. Y. Lu 15610 (PPI); Kaohsiung city: Tenchih, 28 Jun 2000, C. M. Wang 4321 (TNM); same loc., 21 May 2016, S. Z. Yang 77621 (PPI); Nanfengshan, 18 Dec 1961, J. M. Liao 788 (TAI); Meilan forest road 8.6 km, 1300 m alt., 10 May 1994, T. Y. Liu et al. 419 (HAST); Southern cross highway, 26 Dec 2000, S. Z. Yang 29567 (PPI); Miaoli county: Shihmen, 20 Mar 1997, M. Y. Shen 1557 (TAIE); Nanchuang shenmiku, 24 Mar 2001, J. H. Lii 428 (TAIF); Taian hot-spring, 300–400 m alt., 30 Jun 1992, J. C. Wang et al. 7707 (HAST, PPI); Tahu, 200−400 m alt., 27 Jan 2007, H. Y. hsieh 138 (PPI); Nantou county: Bihhwu, 2 Sep 1993, C. M. Wang W00160 (TNM); Kunglingshan, 1 May 1991, H. Y. Chen et al. 1118 (TNM); Chitou, 15 May 1988, S. L. Shern & H. M. Su 42 (TNM); same loc., 5 Feb 2010, C. T. Chao 1323, 1324 (TCF); Wushe to Aowanda, 20 Mar 2009, C. T. Chao 496, 497, 498 (TCF); Chioufengershan, 14 Apr 2010, C. T. Chao 607, 608, 609, 610 (TCF); Aowanda, 7 Nov 2009, C. T. Chao 1129 (TCF); Fenghuangshan, 1225 m alt., 4 Sep 1997, T. W. Hsu 8806 (TAIE); Ching Shui Kou Treet, 14 Dec 1960, T. C. Huang 1937 (TAI); Shalishien river, 1120 m alt., 15 May 1985, C. I Peng 7775 (HAST); en route from Tungpu Hot Soring to Kuankao, 1300–2600 m alt., 3 Jul 1985, C. I Peng 8070 (HAST); Patungkuan Ancient Trail, 1150 m alt., 13 Aug 2004, C. C. Wu et al. 725 (HAST); Old Chinai Village, 8 Jul 2007, P. F. Lu 14172 (HAST); Fengshan, 1200 m alt., 29 Jun 1996, T. W. Hsu 7650 (TAIE); Chuoshuihsi River valley by the Yunlong Bridge, 1100 m alt., 15 Jan 1003, M. H. Chen et al. 70 (HAST); en route from Hsini to Hoshe, ca. 950−1200 m alt., 6 Aug 1991, W. P. Leu 1073 (PPI); New Taipei City: Yunsen Falls, 26 Aug 1996, K. C. Yang & W. F. Ho 4982 (TNM); Homei, S. F. Huang 4292 (TAI); Fushan, 300–600 m alt., 3 Apr 2002, Z. W. Lee 77 (HAST); Huangdidian, 450–500 m alt., 2 Feb 2003, S. C. Liu et al. 976 (HAST); Pingtung county: Wutoushan Nature Protected Area, 21 Apr 1995, K. Y. Wang et al. 950 (TNM); Chinshuiying, 1200 m alt., 22 Jun 1999, K. F. Chung 1262 (HAST); Tahanshan, 1100−1200 m alt., 2 May 2006, J. C. Wu 55 (PPI); Shishan forest road, 1600−1650 m alt., 22 Jul 2002, G. P. Hsieh 588 (PPI); Chiupaoshan, 1000−1500 m alt., 28 May 2004, S. H. Hueng 42515 (PPI); Itingshan, 1200−1500 m alt., 23 May 2003, G. P. Hsieh 1069 (PPI); Ali, 14 Jul 1989, S. Z. Yang 24872 (PPI); Hsuehyehkenshan, 800−1200 m alt., 17 May 2014, P. H. Chen 369 (PPI); Wutai, 9 May 1986, C. E. Chang & L. C. Chang 106 (PPI); Dangmanushan, 25 Mar 1990, S. Z. Yang & C. G. Lin 23036 (PPI); Jihmushan, 27 Apr 1991, S. Z. Yang 24604 (PPI); Taichung City: Wushihkeng, 10 Oct 1998, C. M. Wang 3689 (TNM); Yuantsuishan-Shaolaishan, 8 Sep 1998, S. T. Chiu 4913 (TNM); Tzuyukuohsiao (an elementary school), 750 m alt., 6 Jun 1995, Y. H. Tseng 535 (TAIE); under Mt. Izawa, 17 Jul 1935, H. Simada SH 606 (TAI); tausaiibunaraha, H. Simada SH 1307 (TAI); Tainan city: Tatungshan, 2 Apr 1993, T. C. Huang & S. F. Huang 15989 (TAI); Kantoushan Hsienkung temple, 500–700 m alt., 12 Jan 1996, C. C. Liao et al. 1737 (HAST); Zengwen reservoir, 800−1100 m alt., 24 Jul 2008, C. C. Hsiao 17 (PPI); Taoyuan city: en route between Kaopo and Tawan, 9 May 1993, W. P. Liu 1744 (TNM); Hsiehwunao, 700–800 m alt., 10 Sep 2002, Z. W. Lee 378 (HAST); Taitung county: Kuzulun-shan, 29 Aug 1957, Kao et T. I. Chuang 1103 (TAI); Litao-Tienlung Bridge, 4 Apr 1987, S. F. Huang et al. 3642 (TAI); Lichia forest road, 1225 m alt., 16 Oct 1997, Y. C. Kao et al. 137 (HAST); Shawushan, 700−800 m alt., 2 Aug 2005, C. F. Chen 1708 (PPI); Chianaimeishan, 500−700 m alt., 20 Aug 2005, Y. J. Lin 35 (PPI); Preserve area of *Juglanscathayensis*, 9 Feb 1993, S. Z. Yang 30044 (PPI).

#### 2. Disporum sessile

Taxon classificationPlantaeLilialesColchicaceae

D. Don ex Schult. var. intermedium (Hara) Y.H.Tseng & C.T.Chao comb. nov.

urn:lsid:ipni.org:names:77195641-1

Figs 7 , 8 南投寶鐸花

D. sessile D. Don *ex* Schult. var. shimadai (Hayata) Hara f. intermedium Hara in Univ. Mus., Univ. Tokyo, Bull. 31:203. 1988. TYPE: Kwarenko, between Tosato and Totokun, 11 Apr. 1940, M. Tagawa 3700 (holotype: TI!, isotype: KYO!)
Disporum nantouense S. S. Ying, Mem. Coll. Argic. Natl. Taiwan Univ. 30(2):59. 1990. Wang, Cytotaxonomy of Liliaceae in Taiwan (II) Polygonateae and Tricyrteae. 44. 1997; Chen et al., Fl. China 22:157, 2000; Ying, Fl. Taiwan 2^nd^ ed. 5:46. 2000. Type: Nantou county, Jenai township, Mt. Hohuanshan, near to Meifeng, alt. 2170 m, 9 Apr. 1989, Ying s.n. (holotype: NTUF), syn. nov.
Disporum taipingense M. N. Tamura & Kawano, nom. nud.

##### Perennial herbs.

Stem erect, 15−45 cm, lower nodes covered with scale leaf, persistent, branched at the upper part, with creeping stolon. Leaf deciduous, simple, alternate, lanceolate to linear-lanceolate, 3-nerved, 5−6 cm long, 2−3 cm wide, apex acuminate, base obtuse, petiole short, 3−5 mm long, green, sometimes reddish, estipule, glabrous. Inflorescences pseudoterminal, solitary to 2−3 flowers, peduncle 3−5 mm, bract absent. Pedicels 1−1.5 cm long, tepals 6, arranged into 2-whorls, spathulate, white, apex green with violet spots, 1.5−2 cm long, 6−8 mm wide, margin papillose, 3-nerved, base spurred, with nectary. Stamens 6, inserted at base of tepals, filaments 1−1.5 cm long, anthers 3 mm long, 2-loculed, longitudinally dehiscent. Ovary superior, 3-loculed, style slender, subglabrous, 1−1.5 cm long, stigma 3-fid, pubescent. Fruits berry, globose, purplish-black when mature. Seeds numerous. 2n = 16

##### Endemic to Taiwan.

Distributed in medium altitudes, ca. 1,500 m to 2,900 m.

##### Additional specimens examined.

Chiayi county: Alishan, near Tziyun temple, 14 Apr 1991, T. Y. Yang et al. 5548 (TNM); Alishan, 9 Jul 1981, C. E. Chang 18237 (PPI); on the road side of Mienyueh railroad, 16 Jul 1986, T. Y. A. Yang 3073 (PPI); Tungpu lodge to Chichung, 2400 m alt., 19 Oct 1987, S. Z. Yang 3767 (PPI); Hsinchu county: Yuanyanghu, 1670 m alt., 28 Apr 1995, K. Y. Wang et al. 1059 (HAST); Hualien county: Mt. Muh-kwa, 24 Jul 1961, M. T. Kao K4146 (TAI); Lanshan, 1 Apr 1994, Y. C. Sun 236 (TAIE); Hoping forest track, 1700−2000 m alt., 24 May 1993, S. F. Huang et al. 5128 (TAI); near Shihtung suspension bridge of Patungkuan ancient trail; 1500−1800 m alt., 7 Apr 2009, J. C. Wu 328 (PPI); Taitung county: Hsiangyang in forest, 2350 m alt., 24 Jul 1988, C. I Peng et al. 11876 (HAST); Ilan county: Mt. Taipingshan, 1970 m alt., 3 Apr 1986, T. C. Huang 10813 (TAI); Nan-shan to Chi-li-ting, 1200−1400 m alt., 20 Aug 1969, C. C. Hsu 5834 (TAI); Mt. Nanakotaizan, 14 Jul 1937, SH1330 (PPI); Kaohsiung city: Kuaiku, 5 May 2006, C. M. Wang et al. 8857 (TNM); Chungtzukuan, 20 Dec 2000, S. Z. Yang 25492 (TAIF); Tienchi to Chungchikuang trail, 2000−2400 m alt., 14 Jul 2000, C. C. Hsu 56 (PPI); Nantou county: Tienchi, 16 Jun 1996, S. T. Chiu et al. 3363 (TNM); Tatachiaanbu to entrance of Yushan, 23 Jul 2009, C. T. Chao 838 (TCF); highway no. 18, Tatachia to Shihshan, 24 Jul 2009, C. T. Chao 857 (TCF); Yunhai, 8 May 2004, T. Y. A. Yang 16440 (TNM); May-fong, 17 Apr 1980, Ou & Kao 9316 (TAI); Kun-yang to Yuan-feng, 2700−3000 m alt., 9 Apr 1988, J. C. Wang 5091 (TAI); New Taipei city: Peichatienshan, 26 Feb 2002, S. W. Chung 5134 (TAIF); Pingtung county: en route to Tawushan, 1600−2100 m alt., 16 Jul 1988, T. C. Huang et al. 13618 (TAI); Peitawushan, 1500−1800 m alt., 5 Apr 1997, D. W. Liu 372 (PPI); same loc. 2050−2100 m alt., 29 May 1997, P. F. Sun 56 (PPI); Taichung city: Chinshan-Paikushan, 1 May 1992, H. M. Chang Hunter 14 (TNM); Nankotaizan, Kirittoi to Ekizyu no aida, 8 Jul 1937, H. Simada SH 1308 (TAI); Taitung county: Kuei-hu, 1600 m alt., 29 Jul 1967, C. C. Hsu 3362 (TAI); Hsiangyang, 2200 m alt., 2 Apr 1996, T. Y. Aleck Yang et al. 6451 (PPI, TNM); Chinlunhsi, 1400−1700 m alt., 10 Mar 2007, J. J. Chen 360 (PPI); Taoyuan city: Paling–Lalashan, 17 Apr 1986, T. Y. Yang et al. 2913 (TNM); Lalashan, 10 Apr 2002, S. C. Wu 2608 (TAI);

**Figure 7.**
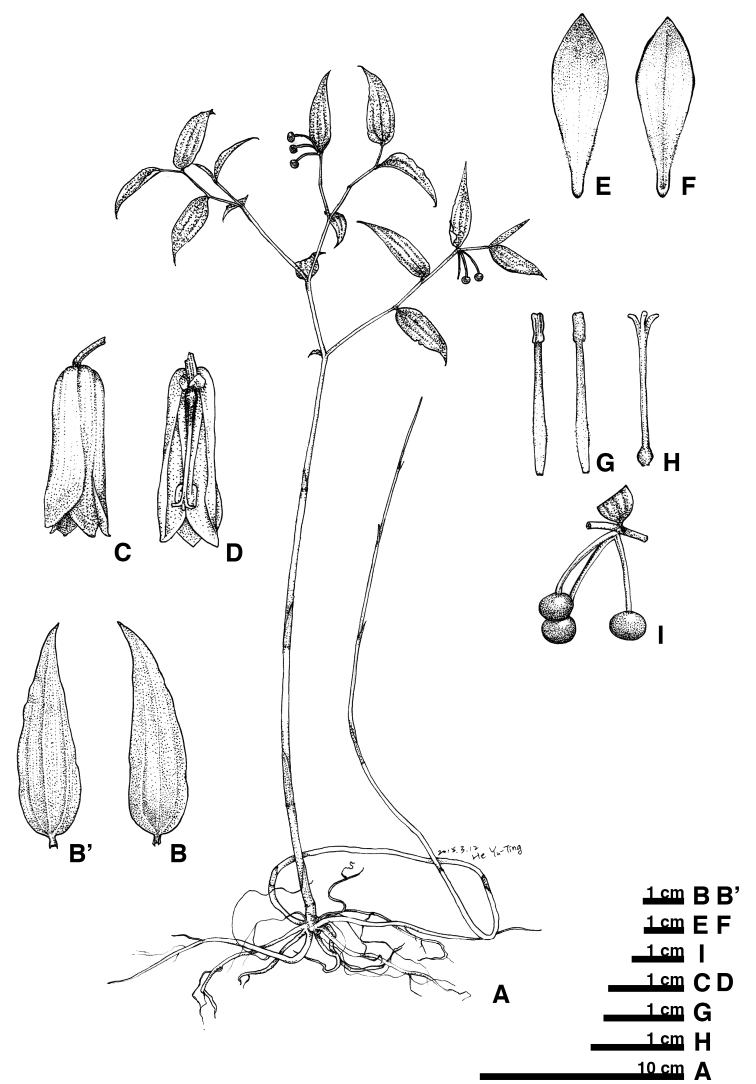
Disporumsessilevar.intermedium. **A** habit **B** leaf adaxial surface **B**’ leaf abaxial surface **C** flower **D** flower section (with tepals and stamen removed) **E** tepal outer surface **F** tepal inner surface **G** stamen **H** pistil **I** fruit.

**Figure 8.**
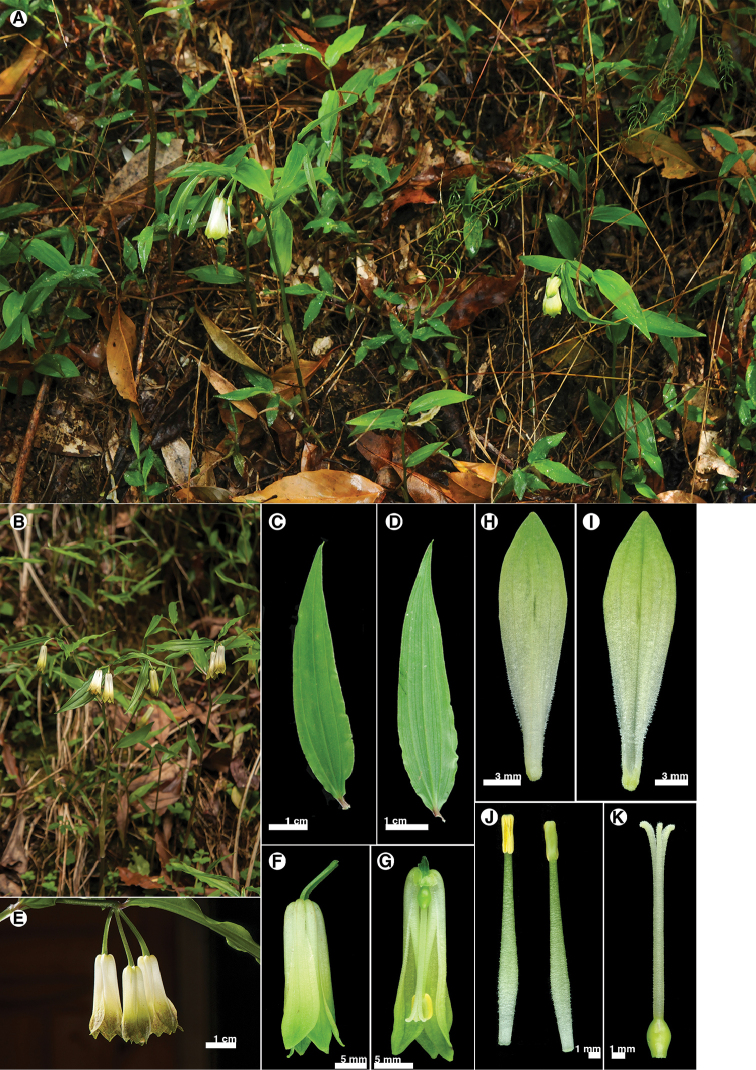
Disporumsessilevar.intermedium. **A** habitat **B** habit **C** leaf adaxial surface **D** leaf abaxial surface **E** inflorescence **F** flower **G** flower section (with tepals and stamen removed) **H** tepal outer surface **I** tepal inner surface **J** stamen **K** pistil.

#### 3. Disporum shimadae

Taxon classificationPlantaeLilialesColchicaceae

Hayata, J. Coll. Sci. Imp. Univ. Tokyo 30(1): 367. 1911

山寶鐸花 Figs 9 , 10

Disporum shimadae Hayata, J. Coll. Sci. Imp. Univ. Tokyo 30(1): 367. 1911. as ‘shimdai’. Hayata, Gen. Ind. 85, 1917; Sasaki, List Pl. Form. 106. 1928; Masamune and Simada, Short Fl. Form. 269. 1936; Masamune, List Vasc. Pl. Taiwan. 132. 1954; Chao et al., Bot. Bull. Acad. Sin. New Series 4(2):81; Ying, The Liliaceae of Taiwan. 24. 1969; Liu & Ying, Fl. Taiwan. 5:54. 1978; Ying, Liliaceae of Taiwan. 28. 1990; Wang, Cytotaxonomy of Liliaceae in Taiwan (II) Polygonateae and Tricyrteae. 46. 1997; Chen et al., Fl. China 22:158, 2000; Ying, Fl. Taiwan 2nd ed. 5:46. 2000; Boufford et al., Fl. Taiwan 2nd ed. 6:111. TYPE: Kelung, Masoku, 6 Mar. 1908, T. Kawakami & Y. Shimada 4311. (isotype: TAI!)
Disporum sessile (Thunb.) D. Don *ex* Schult. var. shimadae (Hayata) Hara, Univ. Mus., Univ. Tokyo, Bull. 31:202 1988.

##### Perennial herbs.

Stem erect, 15−45 cm, covered with persistent scale leaf at lower nodes, branch at the upper part. Leaf deciduous, simple, alternate, lanceolate, 4−10 cm long, 2−4 cm wide, apex acuminate, base obtuse, margin entire, 3-nerved at the base, petiole short, 3−5 mm long, glabrous, estipulate. Inflorescences pseudoterminal, solitary to 3−5 flowers fascicled, peduncle short, 3−5 mm long, bract absent. Tepals 6, arranged into 2-whorls, spathulate, 2−3 cm long, 5−8 mm wide, the inner surface of basal part and margin papillose, base with a short spur, 1−2 mm long, nectary inside. Stamens 6, inserted at base of tepals, filament often expansion at proximal part, 10−15 mm long, glabrous, anthers 2-loculed, basifixed, 3 mm long, longitudinally dehiscent. Ovary superior, 3-loculed, obovate, style 1−1.5 cm long, stigma 3-lobed, pubescent. Fruits berry, purplish-black, seed numerous. 2n = 14.

**Figure 9.**
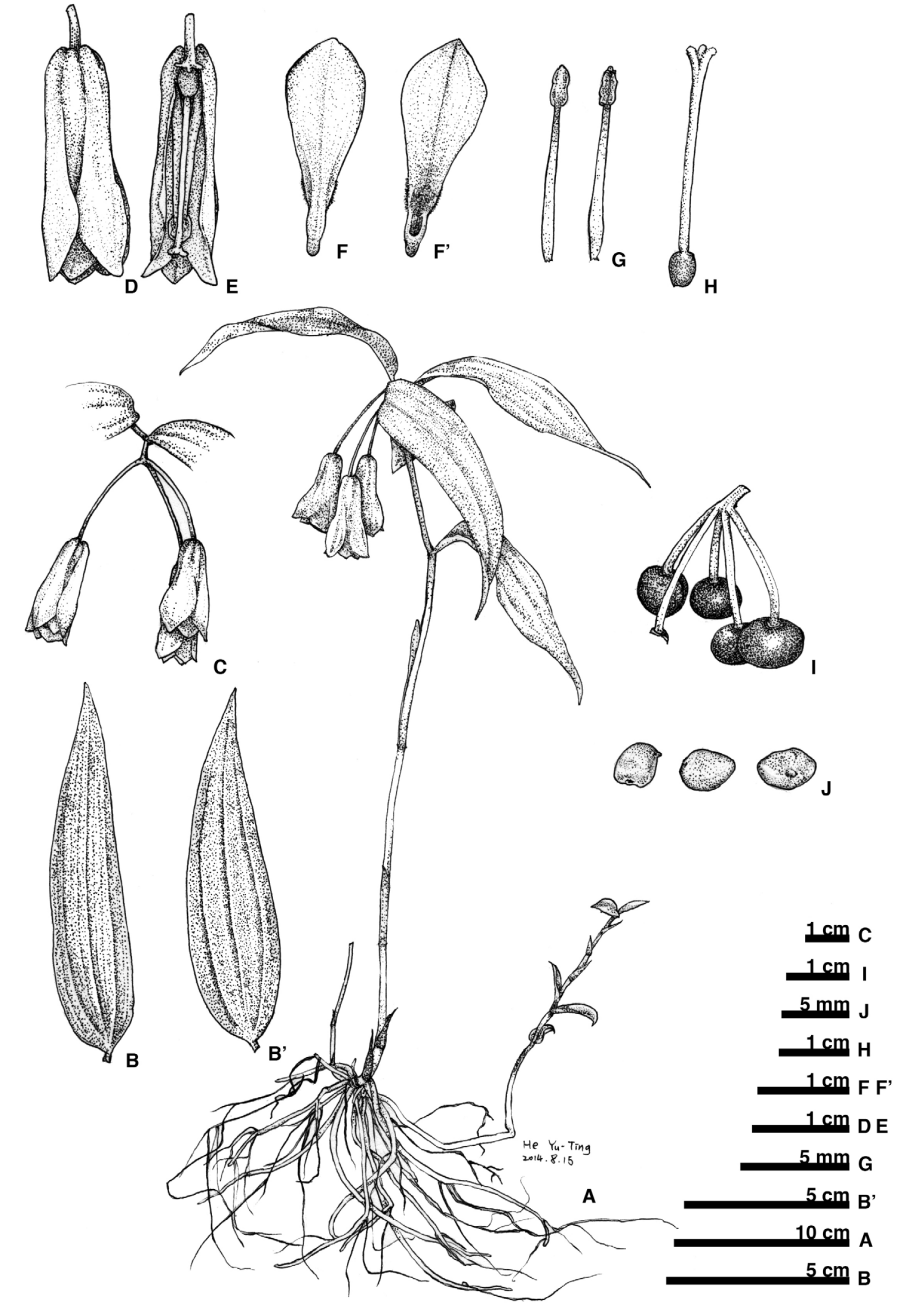
*Disporumshimadae*. **A** habit **B** leaf adaxial surface **B**’ leaf abaxial surface **C** inflorescence **D** flower **E** flower section (with tepals and stamen removed) **F** tepal outer surface **F**’ tepal inner surface **G** stamen **H** pistil **I** fruit **J** seeds.

**Figure 10.**
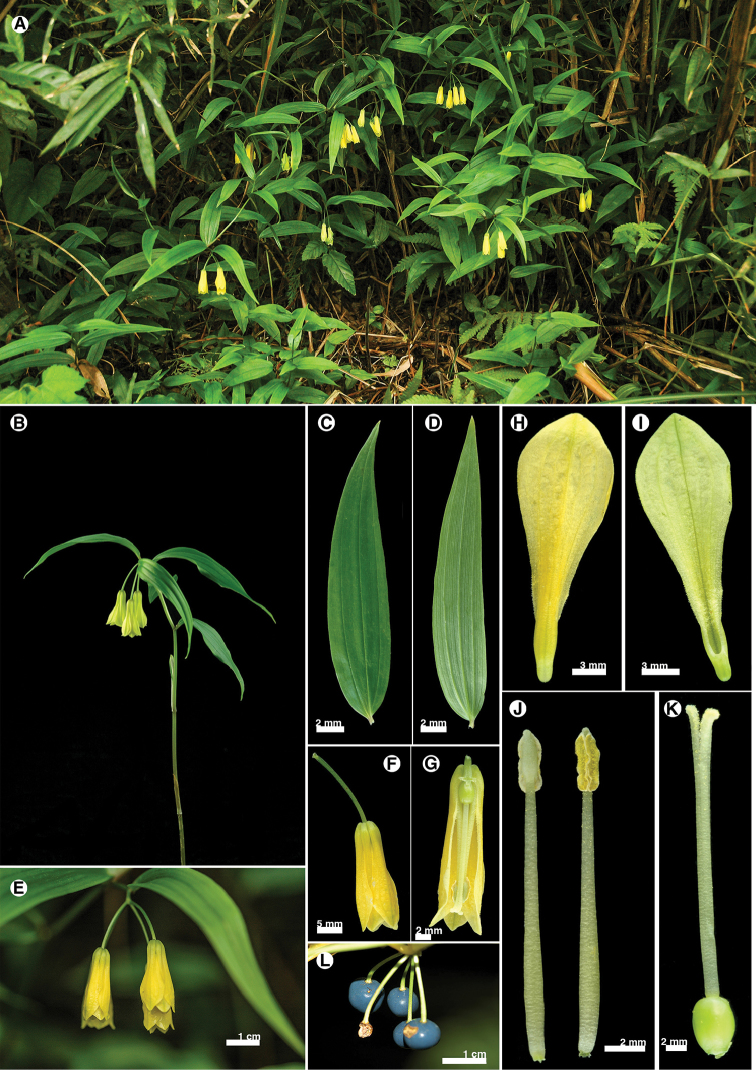
*Disporumshimadae*. **A** habitat **B** habit **C** leaf adaxial surface **D** leaf abaxial surface **E** inflorescence **F** flower **G** flower section (with tepals and stamen removed) **H** tepal outer surface **I** tepal inner surface **J** stamen **K** pistil **L** fruits.

##### Endemic to Taiwan.

Distributed in north-eastern part, from seashore to low altitude mountains.

##### Additional specimens examined.

Ilan county: Tali, 20 Apr 1985, W. S. Tang 1324 (TAI); Kueishan island, 31 May 2003, S. T. Chiu et al. 7542 (TNM); Keelung city: Chyngrenhu, 16 May 2007, T. Y. A. Yang et al. 19220 (TNM), Masu, 6 Mar. 1908, T. Kawakami & S. Simada 4311 (Isotype! TAI); Tawulun Fort, 3 Nov 2009, C. T. Chao 1108, 1111 (TCF); Hoping Island, 12 Mar 1963, S. Y. Lu 643 (TAIF); same loc., 15 Mar 1986, S. Z. Yang 2443 (PPI); Paimiweng Fort, 50 m alt., 4 May 1992, C. C. Liao with C. C. Wang 260 (HAST); New Taipei city: Hsiaotzushan, 21 Mar 1995, H. Y. Shen et al. 560 (TNM); Tatun main peak, 6 Mar 2009, 460, 462, 463, 464, 465 (TCF); Shumeiping, 2 Mar 2002, T. Y. A. Yang et al. 14539 (TNM); Lungtung, 3 Mar 2002, T. Y. A. Yang et al. 14553 (TNM); Dapishan, 23 May 2003; S. C. Liu et al. 1203 (TNM); Homei, 3 m alt., 16 Jun 2004, Y. H. Tseng 3786 (TAIE); Huangdieden shan, 560 m alt., 2 May 1992, L. F. Huang et al. 29 (TAI); Wututan, 22 Aug 2001, H. L. Chiang 2707 (TAIF); Santiaochiao, 28 Feb 2007, F. C. Kuo 29 (TAIF);

## Supplementary Material

XML Treatment for Disporum kawakamii

XML Treatment for Disporum sessile

XML Treatment for Disporum shimadae
